# A systematic review of randomised controlled trials in rheumatoid arthritis: the reporting and handling of missing data in composite outcomes

**DOI:** 10.1186/s13063-016-1402-5

**Published:** 2016-06-02

**Authors:** Fowzia Ibrahim, Brian D. M. Tom, David L. Scott, Andrew Toby Prevost

**Affiliations:** Academic Department of Rheumatology, Faculty of Life Sciences and Medicine, King’s College London, Weston Education Centre, 10 Cutcombe Road, London, SE5 9RJ UK; MRC Biostatistics Unit, Cambridge Institute of Public Health, Cambridge, UK; Imperial Clinical Trials Unit, Imperial College London, Stadium House, 68 Wood Lane, London, W12 7RH UK

**Keywords:** RA, Composite outcomes, Missing data, Imputation, Sensitivity analysis

## Abstract

**Background:**

Most reported outcome measures in rheumatoid arthritis (RA) trials are composite, whose components comprise single measures that are combined into one outcome. The aims of this review were to assess the range of missing data rates in primary composite outcomes and to document the current practice for handling and reporting missing data in published RA trials compared to the Consolidated Standards of Reporting Trials (CONSORT) recommendations.

**Methods:**

A systematic search for randomised controlled trials was conducted for RA trials published between 2008 and 2013 in four rheumatology and four high impact general medical journals.

**Results:**

A total of 51 trials with a composite primary outcome were identified, of which 38 (75 %) used the binary American College of Rheumatology responder index and 13 (25 %) used the Disease Activity Score for 28 joints (DAS28). Forty-four trials (86 %) reported on an intention-to-treat analysis population, while 7 trials (14 %) analysed according to a modified intention-to-treat population. Missing data rates for the primary composite outcome ranged from 2–53 % and were above 30 % in 9 trials, 20–30 % in 11 trials, 10–20 % in 18 trials and below 10 % in 13 trials. Thirty-eight trials (75 %) used non-responder imputation and 10 (20 %) used last observation carried forward to impute missing composite outcome data at the primary time point. The rate of dropout was on average 61 % times higher in the placebo group compared to the treatment group in the 34 placebo controlled trials (relative rate 1.61, 95 % CI: 1.29, 2.02). Thirty-seven trials (73 %) did not report the use of sensitivity analyses to assess the handling of missing data in the primary analysis as recommended by CONSORT guidelines.

**Conclusions:**

This review highlights an improvement in rheumatology trial practice since the revision of CONSORT guidelines, in terms of power calculation and participant’s flow diagram. However, there is a need to improve the handling and reporting of missing composite outcome data and their components in RA trials. In particular, sensitivity analyses need to be more widely used in RA trials because imputation is widespread and generally uses single imputation methods, and in this area the missing data rates are commonly differentially higher in the placebo group.

**Electronic supplementary material:**

The online version of this article (doi:10.1186/s13063-016-1402-5) contains supplementary material, which is available to authorized users.

## Background

Rheumatoid arthritis (RA) is a chronic autoimmune inflammatory disease of unknown aetiology. It is challenging to diagnose RA because of the variability in the disease expression [1–3]. In RA, most reported outcome measures are composites, with the Disease Activity Score for 28 joints (DAS28), a continuous measure of current status of disease activity, and the American College of Rheumatology (ACR) response criteria, a binary indicator of disease activity change over time, being the most commonly used. The ACR response criteria are defined as 20 % (ACR20), 50 % (ACR50) and 70 % (ACR70) improvement in five of the seven measures [4, 5].

A composite endpoint or outcome comprises several single endpoints that are combined into a single outcome. The use of composite endpoints has been discussed extensively in the trial literature; for example, they are used as time-to-event endpoints in cardiovascular, cancer, diabetic and HIV studies [6, 7], where the composite might involve binary variables which combine mortality with non-fatal endpoints such as hospitalisation and cardiac arrest in chronic heart patients [8]. The advantages of using composite outcomes are statistical efficiency and increased precision of risk ratios and other parameter estimates, arising from a larger event rate, and hence a smaller sample size needed when designing the trial [9]. An article by Senn and Julious criticised the use of composite response measures, as they argued that their use should be carefully thought through and accompanied by consideration of their components [10].

Randomised controlled trials (RCTs) are the gold standard study design for evaluating treatment efficacy. Trials with measurements made on the same patient repeated over time nearly always have an outcome where patients have missing values at the end of follow-up. A missing value is an observation that was intended to be collected from a study subject but for a variety of reasons was not collected [11]. The presence of missing data in trials leads to a loss of statistical power to detect effects through a reduction in the size of the analysed sample, when imputation is not used. In addition, the remaining analysed sample may no longer be representative of the recruited sample, which may introduce bias into the treatment estimates. For example, in two-arm trials, these losses and biases can occur differentially in each arm and for reasons connected with changing disease outcome, thereby increasing the potential for incorrect/misleading conclusions from these randomised comparisons if the missing data is inappropriately handled.

A survey of RCTs in all medical fields in four major medical journals in 1999 found that a quarter of trials had more than 10 % of responses missing for the primary outcome [12]. A similar review in 2004 [13] found that 89 % of trials had reported partially missing data, meaning that there is some but not all data available for the individual. In addition, the review showed an unexpectedly high use of overly simple methods for handling missing data which ignored the partially available data. Furthermore, 79 % of trials did not report a sensitivity analysis as recommended by the Consolidated Standards of Reporting Trials (CONSORT) statement [14, 15].

This paper was motivated by the current practice for handling and reporting missing data in RA trials, which has typically involved the use of single imputation methods that have become outdated, e.g. the single imputation method using last observation carried forward (LOCF). Moreover, non-responder imputation (NRI) is another single imputation method, which assigns a subject with a missing binary or categorical outcome as if they are a non-responder. NRI assumes that missing values are treatment failures, and this assumption is unquestioned unless a sensitivity analysis is additionally undertaken in order to explore the impact on the results. The aims of this review were to assess the range of missing data rates in primary composite outcomes and to document the current practice for handling and reporting missing data in published RA trials compared to CONSORT recommendations.

## Methods

### Data sources and search strategy

Trials were identified by searching PubMed and other resources (hand searched individual journal websites, Web of Science, Cochrane Central Register of Controlled Trials and Google Scholar). The Preferred Reporting Items for Systematic reviews and Meta-Analyses (PRISMA) guidelines were followed for reporting the review methodology [16]. The search terms are given in Additional file 1: section A.

### Selection of studies

Studies were included in the review if they met the inclusion criteria: phase 3; double blinded RCT conducted in adults with RA; English language papers published between January 2008 and December 2013; published in four rheumatology journals (*Annals of the Rheumatic Diseases, Arthritis & Rheumatism, Arthritis Research & Therapy* and *Rheumatology*); and four high impact factor general medical journals (*Lancet, New England Journal of Medicine, Journal of the American Medical Association* and the *British Medical Journal*); and a composite outcome measure was reported as the primary outcome.

### Data extraction

Data extracted from the papers included the following: year of publication, journal, source of funding, primary and secondary outcomes, trial design; sample size calculation and whether this calculation included a dropout rate; amount of missing information (proportion of missing outcome data in each treatment group after randomisation); method of dealing with missing primary outcome data; analysis population (intention-to-treat (ITT), modified ITT, per protocol, complete or available cases); statistical method used to analyse the primary outcome; sensitivity analyses; participant flow diagram (e.g. number randomised in each group, number included in the ITT analysis, number completed and number of withdrawals or lost to follow-up) and study follow-up time. ITT is an approach used to analyse trial data, where subjects are analysed in their original randomised group irrespective of whether they received the intervention or not.

### Statistical analysis

Data manipulation and analyses were carried out in Stata (version 13.0, StataCorp, College Station, TX). The proportion of missing primary outcomes at the primary time point was defined as: one minus the number of patients who completed the trial divided by the number of patients in ITT analysis. The treatment group that we presented here represents the combined active treatment groups in each trial; e.g. if a trial had three arms of which two arms were active treatment and one a placebo, then the numbers in the two active treatment groups were combined. To estimate the differential rate of missing primary outcome data, the relative rate of missing data was defined to be the rate of missing primary outcome in the placebo group relative to that in the treatment group. Summary statistics were presented to describe the characteristics of the studies included in the review. Chi-square or Fisher’s exact tests were used to compare between the categories.

## Results

### Study selection

**Fig. 1:**
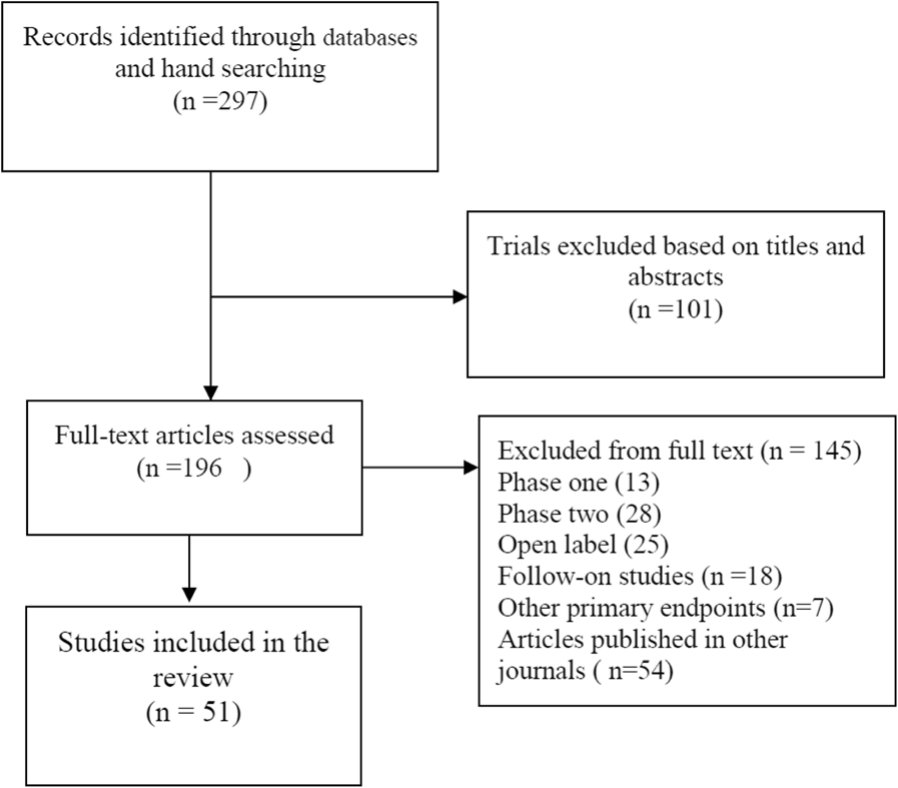
PRISMA flow diagram for study selection

The initial database search identified 297 unique studies, from which 196 were selected for a full text examination. A further 145 articles were excluded, leaving 51 papers (see Fig. 1). The majority of exclusions at this stage were made for studies not being in phase 3 or published in other journals.

### Characteristics of included studies

Of the 51 trials published between 2008–2013, 23 (45 %), 11 (22 %) and 17 (33 %) were published in the periods 2008–2009, 2010–2011 and 2012–2013, respectively (see Table 1). Fifty of the 51 trials were of parallel design (98 %), and of these, 26 (52 %) were two-arm treatment comparisons, 14 (28 %) three-arm comparisons and the remaining 10 (20 %) had more than three arms.

**Table 1:** Characteristics of the trials included in the review

Characteristic	Number of trials (*N* = 51)
Publication year	
2008	12 (24 %)
2009	11 (22 %)
2010	7 (14 %)
2011	4 (9 %)
2012	8 (16 %)
2013	9 (18 %)
Journals	
Rheumatology journals	
*Annals of the Rheumatic Diseases*	19 (37 %)
*Arthritis & Rheumatism*	17 (33 %)
*Rheumatology*	5 (10 %)
*Arthritis Research & Therapy*	1 (2 %)
General medical journals	
*Lancet*	7 (14 %)
*New England Journal of Medicine (NEMJ)*	2 (4 %)
*British Medical Journal (BMJ)*	0 (0 %)
*Journal of the American Medical Association (JAMA)*	0 (0 %)
Funding	
Pharmaceuticals	47 (92 %)
Academic/charity	4 (8 %)
Subjects	
Randomised subjects per trial, median (IQR)	159 (102–249)
Mean age in years, median (IQR)	52 (50–54)
Mean disease duration in years, median (IQR)	7.6 (2.3–9.1)
Follow-up time in weeks, median (IQR)	24 (14–26)
12–23 weeks	14 (27 %)
24 weeks	24 (47 %)
>24 weeks	13 (26 %)

The binary ACR20 responder index was the most frequently reported primary composite outcome in 35 trials (69 %), followed by 12 trials (24 %) reporting DAS28-ESR as the primary outcome, one trial reporting DAS28-CRP, and three (6 %) reporting the ACR50 responder index. The DAS28 measures are continuous composites, and the ACR indices are binary composites.

The median trial duration was 24 weeks (interquartile range IQR: 14–26 weeks). The mean age and disease duration of the participants in the trials at baseline ranged from 46.4 to 60.0 years, and 0.2 to 16.9 years, respectively.

### CONSORT flow diagram

A participant flow diagram was reported in 43 trials (84 %). There was a rise in the use of the recommended flow diagram from 74 % in 2008–2009 (pre CONSORT, revised in 2010) to 93 % in 2011–2013 (post CONSORT). All nine of the trials published in the general medical journals reported a flow diagram. For those published in the specialist rheumatology journals, eight trials did not report a flow diagram.

### Sample size calculation

**Fig. 2:**
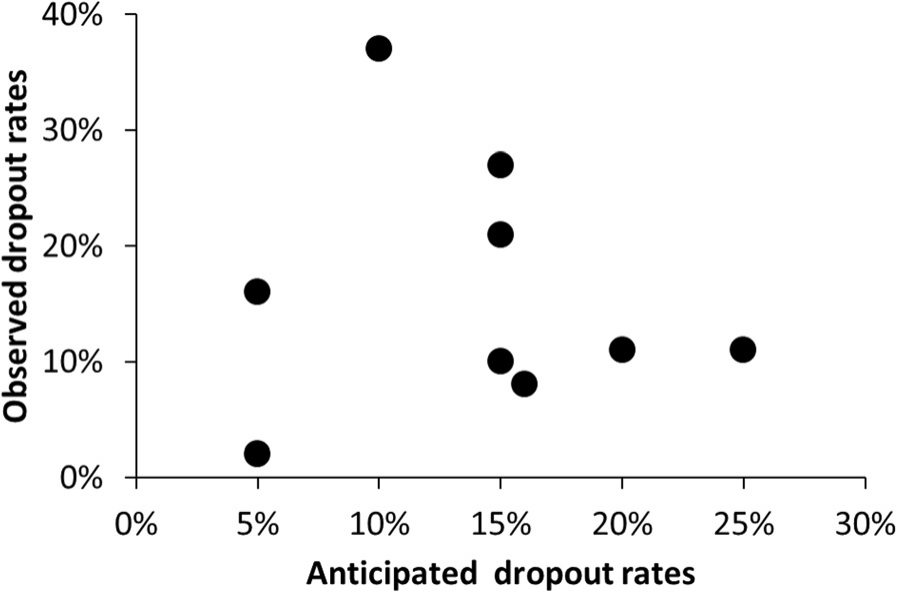
Observed and anticipated dropout rates. Each dot represents one trial; only 9 trials included dropout rate in the sample size calculations

Forty-three trials (84 %) reported sample size calculations. Of these, 9 (21 %) trials included an allowance for dropout in their power calculation. This dropout rate ranged from 5–25 %, and 4 out of 9 trials (44 %) underestimated the dropout rate (Fig. 2). The proportion of trials that reported a sample size calculation was similar in trials published pre and post CONSORT (87 % versus 82 %, *p* = 0.715). A similarly high proportion, 37 (73 % of) trials, reported both a sample size calculation and a participant flow diagram.

The reporting of a sample size calculation and participant flow diagram shows a direction of improvement with the introduction of the CONSORT statement (Table 2), although these improvements were not significant at the 5 % level. In the pre CONSORT revision period, the number of trials reporting both a sample size calculation and a participant flow diagram were 16 of 23 trials (70 %), and in the post CONSORT period the proportion was slightly higher, 21 of 28 (75 %).

**Table 2:** Conformity with missing data-related CONSORT items by year of trial publication

	Year of publication			
CONSORT items	2008–2009 *n* = 23	2010–2013 *n* = 28	*P* value	All *N* = 51
Sample size statement given				
Yes	20 (87 %)	23 (82 %)	0.638	43 (84 %)
Dropout planned for^a^	4 (20 %)	5 (22 %)	0.965	9 (21 %)
Flow diagram given				
Yes	17 (74 %)	26 (93 %)	0.064	43 (84 %)
Sample size and flow diagram given	16 (70 %)	21 (75 %)	0.689	37 (73 %)
ITT analysis stated	20 (87 %)	24 (86 %)	0.898	44 (86 %)
Used sensitivity analysis				
Yes	5 (22 %)	9 (32 %)	0.4261	14 (27 %)

^a^A percentage of anticipated dropouts were included in the power calculations

### Intention-to-treat analysis

In most trials, it was stated that the primary analyses were based on the ITT population. Forty-four (86 %) of the trials reported that ITT analyses were used, while the remaining 7 (14 %) of the trials analysed the primary outcome data according to a modified ITT population, which was defined as all randomised patients who had taken at least one dose of study medication and had a baseline and at least one other visit; see Additional file 1: Table S1.

### Extent of missingness in the primary composite outcomes (reporting of missing data)

Missing values were present in primary composite outcomes for all of the 51 trials. The median missing primary composite outcome rate was 17 % (IQR 10–25 %) with a wide range from 2.1–52.7 %. Typically 17 % of the primary composite outcome data in ITT analyses were imputed data, and this was considerably higher for some trials. The rate of imputed missing primary outcome was >30 % in 9 trials (18 %), >20–30 % in 11 trials (22 %), 10–20 % in 18 trials (35 %), and <10 % in 13 trials (25 %).

### Imputation details of the primary composite endpoints (missing data handling)

In the 38 trials with binary outcomes with imputation of the whole composite, NRI was used in 29 trials (76 %), and 6 trials (16 %) used LOCF. Moreover, in 3 trials no imputation method was used, although these trials had 10–13 % of missing data in the primary outcome data. Similarly, in the 13 trials that reported DAS28 (which is a continuous composite containing mixed continuous and binary constituents), both of these single imputation methods were prominent, with a greater number, 9 (69 %), using the NRI than LOCF, 4 (31 %).

Some trials used both of these imputation methods in their outcomes. For example, out of the 38 trials which reported using NRI, 23 (61 %) also used LOCF. One trial used both LOCF and multiple imputation (MI). MI is a general statistical method to analyse incomplete data. It attempts to impute missing information by repeating the imputation process multiple times, with each imputation consisting of a value randomly drawn from a distribution of likely values determined from the observed data [11].

Four trials reported using the LOCF method to impute the joint count component only [17–20]. A further two trials reported imputing the components of the ACR core set using LOCF [21, 22] while reporting the use of NRI to impute patients missing the whole primary endpoint (ACR20 responder index).

Simple univariate methods were used to analyse primary composite binary outcomes such as the Cochran–Mantel–Haenszel test in 24 trials (47 %), a simple descriptive comparison (i.e. Fisher’s exact or the chi-square test) in 15 trials (29 %) and the binomial comparison in 4 trials (see Table 3). Repeated data of the outcome measured over time was only used in one trial, using a mixed model analysis [23].

**Table 3:** Test statistics used to analyse the primary outcome

Test statistics	Number (%) *n* = 51
Cochran–Mantel–Haenszel	24 (47 %)
χ^2^/Fisher’s exact	15 (29 %)
Logistic regression	5 (10 %)
Binomial comparison	4 (8 %)
ANOVA/ANCOVA	2 (4 %)
Mixed model analysis	1 (2 %)

*ANOVA* analysis of variance, *ANCOVA* analysis of covariance

### Differential rate of missing outcomes between treatment and placebo groups

**Fig. 3:**
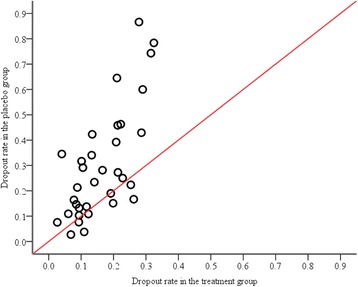
Differential rate of missing data for the primary composite outcome in placebo controlled trials. The line of equality represents no difference between groups; each dot represents one trial. There are more data points above the line, which indicates that trials generally have a higher rate of dropout in the placebo group than in the treatment group

Of the 51 trials, 34 (67 %) were placebo controlled. There were notable differences in the rate of missing primary composite outcome data between treatment and placebo groups. In the treatment group the median rate was 14 %, whereas in the placebo group the rate was 24 %. A formal comparison of treatment and placebo groups in these 34 trials provided a relative rate of 1.61 (95 % CI: 1.29, 2.02), indicating that the rate of missing data in primary composite outcomes was on average 61 % higher in relative terms in the placebo group compared to the treatment group (Fig. 3). For the remaining 17 trials that were not placebo controlled, the median rate of dropout was 11 %, which is lower than that for the placebo controlled trials. Table 4 shows the relative rate of missing primary composite outcome data by length of follow-up. The effect of a higher rate of placebo group dropout was observed to be a little stronger in trials with 6 months or longer follow-up (trend test *p* = 0.296).

**Table 4:** Rates of missing data in placebo relative to treatment group in placebo controlled trials (*n* = 34)

Months of follow-up	Relative rate
	Mean (95 % CI)
3 months (*n* = 12)	1.36 (0.80, 2.29)
6 months (*n* = 16)	1.72 (1.26, 2.35)
12 months (*n* = 5)	1.78 (1.19, 2.64)
Total (*n* = 34)^a^	1.61 (1.29, 2.02)

17 trials were excluded due to not having a placebo group; trend test *p* value = 0.296

*CI* confidence intervals

^a^The total number of trials includes one trial with 24 months follow-up

### Sensitivity analysis for assessing the handling of missing data

Of the 51 trials, 14 (27 %) reported the use of a sensitivity analysis (see Additional file 1: Table S1 for full descriptions). Sensitivity analyses were used in 38 % (11/29) of trials with more than 15 % missing data in the primary outcome, and used in 14 % (3/22) of trials with less than 15 % missing data.

### Missing data mechanism

There was no discussion of the impact that the handling of the missing data might have had on the primary analysis in any of the trials. An initial detail is to report the so-called missing data *mechanisms*, which are used to describe the assumed relationship between the observed data and the missing data. Amongst the trials in this review, only four mentioned the missing data mechanism, and further details were given for two of these in supplementary material [23–26].

## Discussion

Our results show that the trials in this review had a wide range of rates of missing values in the primary composite outcome. The methods that were used to handle and report missing data were not clearly reported. This was particularly the case for the components of the composite. Often, the reporting of these trials did not follow the recommendations set out in the CONSORT guidelines [14, 15]. The majority of trials provided a flow diagram that contained the number of patients used in the analysis for the overall primary composite outcome. While the diagram is informative, it does not include all the information required. For example, subjects might be still in the trial and have a missing measurement but contribute to the primary time point. Furthermore, the way current trials are reported often means that we know how many subjects are missing any data within the overall composite, but not for which of the individual components it is missing.

The level of missing composite outcome data varied from 2–53 %, and, typically, 17 % of primary composite data in ITT analyses were imputed. The extent of missing data in the components of the composite was unknown. This is crucial information, as the individual components might have different amounts of missing data or have some partial available data which could be used to inform the primary outcome. We found differential rates of missing data between treatment and placebo groups in the placebo controlled trials; the rate was on average 61 % times higher in the placebo group compared to the treatment group.

Most of the trials (43/51) provided a power calculation. However only 9 trials included a dropout rate in their power calculation, and 44 % of these trials underestimated the dropout rate that was allowed for. The remaining 34/43 trials did not allow for any dropout, and yet the median missing primary outcome was high, 18 % (12–25 %), which clearly reduces the power of the study, although the use of single imputation could unsatisfactorily be argued to overcome the loss of sample size. Moreover, some trials reported analysis of a modified ITT population, which further reduces the numbers from those in an ITT analysis. Although restricting analysis to the modified ITT is not expected to introduce bias per se, it will most likely reduce the power of the trial, may introduce bias and could be contrary to the spirit of the ITT principle [27].

As recently as 2013, methods known to lead to bias trial results, i.e. LOCF and NRI, continue to be used. Furthermore, articles that were published in 2013 (*n* = 10) reported the use of both methods in the primary analyses. Single imputation methods, as used widely in this area, are inappropriate for handling missing outcome data in trials, as they underestimate the true variability in the data [28]. In addition, if missing data are inadequately handled in primary analyses, there is the potential for misleading conclusions due to inadequately capturing the true variability in the data. However, there are other imputation methods that are highly recommended by experts in the field of missing data, i.e. multiple imputations [11, 29, 30].

Some trials in this review reported using LOCF to impute missing joint count constituents of composite outcomes, while using NRI for subjects missing the whole primary composite outcome. It was surprising that most of the trials did not discuss the missing data mechanism. The choice of the method to handle missing data relates to an assumption of missingness [12] and needs to be discussed in sufficient detail for transparency. It is also important to explore the pattern and mechanism of missingness and to report these. In our results, 6 trials mentioned the missing data mechanism.

We also identified a low proportion of trials reporting a sensitivity analysis, i.e. 14 (27 %). A sensitivity analysis, in addition to the main analysis, is generally recommended in order to assess the robustness of the study findings to plausible assumptions made about the missing data [29, 31] and to increase confidence in the validity and generalisability of the results. Moreover, the subject’s primary outcome data were imputed typically for 61 % more subjects in the placebo group than in the active treatment group, without challenge to the assumptions.

Our results have similarities with other studies that report missing data in primary outcome measures. A recent meta-analysis by Hewitt et al. [32] of quality of life outcome in musculoskeletal conditions found that attrition rates of these trials ranged from 4–28 %. It also showed a differential rate of missing data between treatment and control groups, which varied from 1–14 % in the treatment group and 3–25 % in the control group, respectively. Our study also shows similar results to other reviews that were carried out a decade ago. A study by Baron et al. [33] in rheumatic disease found high percentages of missing data on structural outcomes, and these trials did not adhere to the principle of ITT analysis. The review of Wood et al. [13] also found that inappropriate methods were used to handle missing primary outcome data and showed a similarly low percentage of trials reporting sensitivity analyses compared to our results (21 % versus 27 %).

Our study has some limitations. First, we searched four rheumatology and four high impact factor general medical journals, which excludes RA trials that are published elsewhere or in lower impact factor journals. Secondly, we excluded 54 articles that were published in other journals that did not endorse the CONSORT statement, although the majority of these articles did not report a composite outcome at the primary time point. Thirdly, we restricted our search to trials that reported a composite outcome as the primary endpoint. It is true that the space limitations in journals for articles are limited, and therefore some discrepancies may exist between the actual method used and the methods that were reported. Finally, these are RA disease-specific trials and so are not generalisable to other disease areas where the primary outcomes might not be of composite nature.

This study adds to the volume of the existing evidence of reporting and handling of missing outcome data. However, these findings require further investigation because our understanding on whether to impute the whole composite outcome or the individual components and then calculate the composite is unknown. Furthermore, there are no guidelines on how to handle missing data in composite outcome data that result from derived variables. We therefore designed a simulation study that uses information from these results to answer this crucial question.

## Conclusions

This review highlights improvements in rheumatology trial practice since the revision of the CONSORT guidelines, in terms of reporting the power calculation and participant’s flow diagram. However, there is a need to improve the reporting and handling of missing composite outcome data and their components. In particular, sensitivity analyses need to be more widely used in RA trials because imputation is widespread and with assumptions and variability unchallenged, and missing data rates are differentially higher in placebo groups in this area.

### Recommendations to improve the reporting and handling of missing data in composite outcome data in RA trials

Our recommendations are as follows:

- Include the missing composite and its components during follow-up in a table format that shows the missing proportions in each arm at each time point.
- Choose an appropriate method for handling missing data by considering the range of currently available methods. These include multiple imputation and mixed effects models.
- Discuss the potential missing data mechanism and the observed pattern of missingness to support the chosen methods.
- As recommended in the CONSORT guidelines, conduct a sensitivity analysis to show the robustness of the primary analysis to the assumptions that are made when handling missing data.

## Abbreviations

ACR, American College of Rheumatology; ANCOVA, analysis of covariance; ANOVA, analysis of variance; BMJ, British Medical Journal; CONSORT, Consolidated Standards of Reporting Trials; CRP, C-reactive protein; DAS28, Disease Activity Score for 28 joints; ESR, erythrocyte sedimentation rate; IQR, interquartile range; ITT, intention-to-treat; JAMA, Journal of American Medical Association; LOCF, last observation carried forward; NEJM, New England Journal of Medicine; NRI, non-responder imputation; PRISMA: Preferred Reporting Items for Systematic reviews and Meta-Analyses; RA, rheumatoid arthritis; RCT, randomised controlled trial

## Additional file

Additional file 1: Search terms of electronic databases and detailed characteristics of the studies included in the systematic review. (DOCX 167 kb)

## References

[1] Scott DL, Wolfe F, Huizinga TW (2010). Rheumatoid arthritis. Lancet.

[2] Felson DT, Anderson JJ, Boers M, Bombardier C, Chernoff M, Fried B (1993). The American College of Rheumatology preliminary core set of disease activity measures for rheumatoid arthritis clinical trials. The Committee on Outcome Measures in Rheumatoid Arthritis Clinical Trials. Arthritis Rheum.

[3] Felson DT, Anderson JJ, Meenan RF (1990). Time for changes in the design, analysis, and reporting of rheumatoid arthritis clinical trials. Arthritis Rheum.

[4] Prevoo ML, van 't Hof MA, Kuper HH, van Leeuwen MA, van de Putte LB, van Riel PL (1995). Modified disease activity scores that include twenty-eight-joint counts. Development and validation in a prospective longitudinal study of patients with rheumatoid arthritis. Arthritis Rheum.

[5] Felson DT, Anderson JJ, Boers M, Bombardier C, Furst D, Goldsmith C (1995). American College of Rheumatology. Preliminary definition of improvement in rheumatoid arthritis. Arthritis Rheum.

[6] Wittkop L, Smith C, Fox Z, Sabin C, Richert L, Aboulker JP, et al. Methodological issues in the use of composite endpoints in clinical trials: examples from the HIV field. Clinical trials (London, England). 2010;7(1):19–35. doi:10.1177/1740774509356117. 10.1177/1740774509356117 20156955

[7] Ferreira-Gonzalez I, Busse JW, Heels-Ansdell D, Montori VM, Akl EA, Bryant DM, et al. Problems with use of composite end points in cardiovascular trials: systematic review of randomised controlled trials. BMJ (Clinical research ed). 2007;334(7597):786. 10.1136/bmj.39136.682083.AE PMC1852019 17403713

[8] Cohn JN, Tognoni G (2001). Valsartan Heart Failure Trial I. A randomized trial of the angiotensin-receptor blocker valsartan in chronic heart failure. N Engl J Med.

[9] Quan H, Zhang D, Zhang J, Devlamynck L (2007). Analysis of a binary composite endpoint with missing data in components. Stat Med.

[10] Senn S, Julious S (2009). Measurement in clinical trials: A neglected issue for statisticians?. Stat Med.

[11] Carpenter JR, Kenward MG. Missing data in randomised controlled trials—a practical guide. Birmingham: National Institute for Health Research, Publication RM03/JH17/MK. 2008. http://www.pcpoh.bham.ac.uk/publichealth/methodology/projects/RM03_JH17_MK.shtml. Accessed 30 Apr 2015.

[12] Hollis S, Campbell F (1999). What is meant by intention to treat analysis? Survey of published randomised controlled trials. BMJ (Clin Res Ed).

[13] Wood AM, White IR, Thompson SG (2004). Are missing outcome data adequately handled? A review of published randomized controlled trials in major medical journals. Clin Trials.

[14] Murphy JF (2010). Consort 2010 statement on randomised controlled trials. Ir Med J.

[15] Schulz KF, Altman DG, Moher D, Group C (2010). CONSORT 2010 statement: updated guidelines for reporting parallel group randomised trials. BMJ (Clin Res Ed).

[16] Moher D, Liberati A, Tetzlaff J, Altman DG (2009). Preferred reporting items for systematic reviews and meta-analyses: the PRISMA statement. Ann Intern Med..

[17] Dougados M, Kissel K, Sheeran T, Tak PP, Conaghan PG, Mola EM (2013). Adding tocilizumab or switching to tocilizumab monotherapy in methotrexate inadequate responders: 24-week symptomatic and structural results of a 2-year randomised controlled strategy trial in rheumatoid arthritis (ACT-RAY). Ann Rheum Dis.

[18] Emery P, Keystone E, Tony HP, Cantagrel A, van Vollenhoven R, Sanchez A (2008). IL-6 receptor inhibition with tocilizumab improves treatment outcomes in patients with rheumatoid arthritis refractory to anti-tumour necrosis factor biologicals: results from a 24-week multicentre randomised placebo-controlled trial. Ann Rheum Dis.

[19] Yazici Y, Curtis JR, Ince A, Baraf H, Malamet RL, Teng LL (2012). Efficacy of tocilizumab in patients with moderate to severe active rheumatoid arthritis and a previous inadequate response to disease-modifying antirheumatic drugs: the ROSE study. Ann Rheum Dis.

[20] Smolen JS, Beaulieu A, Rubbert-Roth A, Ramos-Remus C, Rovensky J, Alecock E (2008). Effect of interleukin-6 receptor inhibition with tocilizumab in patients with rheumatoid arthritis (OPTION study): a double-blind, placebo-controlled, randomised trial. Lancet.

[21] Smolen J, Landewe RB, Mease P, Brzezicki J, Mason D, Luijtens K (2009). Efficacy and safety of certolizumab pegol plus methotrexate in active rheumatoid arthritis: the RAPID 2 study. A randomised controlled trial. Ann Rheum Dis.

[22] Weinblatt ME, Bingham CO, Mendelsohn AM, Kim L, Mack M, Lu J (2013). Intravenous golimumab is effective in patients with active rheumatoid arthritis despite methotrexate therapy with responses as early as week 2: results of the phase 3, randomised, multicentre, double-blind, placebo-controlled GO-FURTHER trial. Ann Rheum Dis.

[23] Moreland LW, O’Dell JR, Paulus HE, Curtis JR, Bathon JM, St Clair EW (2012). A randomized comparative effectiveness study of oral triple therapy versus etanercept plus methotrexate in early aggressive rheumatoid arthritis: the treatment of Early Aggressive Rheumatoid Arthritis Trial. Arthritis Rheum.

[24] Emery P, Breedveld FC, Hall S, Durez P, Chang DJ, Robertson D (2008). Comparison of methotrexate monotherapy with a combination of methotrexate and etanercept in active, early, moderate to severe rheumatoid arthritis (COMET): a randomised, double-blind, parallel treatment trial. Lancet.

[25] Gabay C, Emery P, van Vollenhoven R, Dikranian A, Alten R, Pavelka K (2013). Tocilizumab monotherapy versus adalimumab monotherapy for treatment of rheumatoid arthritis (ADACTA): a randomised, double-blind, controlled phase 4 trial. Lancet.

[26] van der Heijde D, Tanaka Y, Fleischmann R, Keystone E, Kremer J, Zerbini C (2013). Tofacitinib (CP-690,550) in patients with rheumatoid arthritis receiving methotrexate: twelve-month data from a twenty-four-month phase III randomized radiographic study. Arthritis Rheum.

[27] White IR, Carpenter J, Horton NJ (2012). Including all individuals is not enough: lessons for intention-to-treat analysis. Clin Trials.

[28] Thabane L, Mbuagbaw L, Zhang SY, Samaan Z, Marcucci M, Ye CL (2013). A tutorial on sensitivity analyses in clinical trials: the what, why, when and how. BMC Med Res Methodol.

[29] Little RJ, D’Agostino R, Cohen ML, Dickersin K, Emerson SS, Farrar JT (2012). The prevention and treatment of missing data in clinical trials. N Engl J Med.

[30] O’Neill RT, Temple R (2012). The prevention and treatment of missing data in clinical trials: an FDA perspective on the importance of dealing with it. Clin Pharmacol Ther.

[31] White IR, Horton NJ, Carpenter J, Pocock SJ (2011). Strategy for intention to treat analysis in randomised trials with missing outcome data. BMJ (Clin Res Ed)..

[32] Hewitt CE, Kumaravel B, Dumville JC, Torgerson DJ (2010). Trial attrition study group. Assessing the impact of attrition in randomized controlled trials. J Clin Epidemiol.

[33] Baron G, Boutron I, Giraudeau B, Ravaud P (2005). Violation of the intent-to-treat principle and rate of missing data in superiority trials assessing structural outcomes in rheumatic diseases. Arthritis Rheum.

