# Supplementary material for: A systematic review of randomised controlled trials in rheumatoid arthritis: the reporting and handling of missing data in composite outcomes
Source: Trials. 2016 Jun 2;17:272. doi: 10.1186/s13063-016-1402-5 (PMC4890523; doi:10.1186/s13063-016-1402-5)
Supplement: Additional file 1: — Search terms of electronic databases and detailed characteristics of the studies included in the systematic review. (DOCX 167 kb) [file 13063_2016_1402_MOESM1_ESM.docx]

**Additional File 1**

Section A: **Search terms**

The following request were performed in PUBMED: ("randomized controlled trial"[Publication Type] OR "randomized controlled trials as topic"[MeSH Terms] OR "randomised controlled trials"[All Fields] OR "randomized controlled trials"[All Fields]) AND ("arthritis, rheumatoid"[MeSH Terms] OR ("arthritis"[All Fields] AND "rheumatoid"[All Fields]) OR "rheumatoid arthritis"[All Fields] OR ("rheumatoid"[All Fields] AND "arthritis"[All Fields])) AND (("2008/01/01"[PDAT] : "2013/12/31"[PDAT]) AND "humans"[MeSH Terms] AND English[lang]).

The search terms for the individual journal websites, Web of Science, Cochrane Central Registry of Controlled Trials and Google scholar were not comprehensive and years were restricted between 2008 and 2013.

These search terms were used:

- ‘randomized controlled trials’ AND ‘rheumatoid arthritis’
- ‘randomised controlled trials’ AND ‘rheumatoid arthritis’
- ‘randomized controlled trials’ AND ‘arthritis rheumatoid’
- ‘randomised controlled trials’ AND ‘arthritis rheumatoid’

**Additional File 1: Table S1: Detailed characteristics of the studies included in the review**

| **Reference**  **(year)** | **Number**  **Randomised**  **/ No. in ITT** | **Number completed**  **the trial** | **CONSORT**  **(flow diagram)** | **Sample size** | **Primary outcome**  **(composite)** | **Population**  **Analysed** | **% missing at primary outcome** | **Method of imputation for primary outcome** | **Method of analysed for primary outcome** | **Sensitivity analysis** |
| --- | --- | --- | --- | --- | --- | --- | --- | --- | --- | --- |
| Alten *et al*.  (2010) [1] | 327/327 | 288 | No | Yes | The primary efficacy end point was the proportion of patients with **ACR20** response at week 12 | ITT | 12% | Patients with missing ACR20 values at week 12 were classified as **non-responders** | Primary efficacy was analysed using the CMH to compare treatment groups | No |
| Braun *et al*. (2008) [2] | 384/383 | 375 | Yes | Yes^¥^ | The primary end point was the percentage of patients with an **ACR20** response at week 24 | ITT | 2% | The last value recorded for the associated efficacy parameter was carried forward (**LOCF).** Only post-baseline values were allowed to be carried forward | CHM chi-square test, with adjustment for study centers was used to compare successful response between treatment arms | No |
| Bruyn *et al*. (2008) [3] | 121/121 | 95 | Yes | Yes | The primary efficacy was the proportion of patients with an **ACR20** response at week 12 | ITT | 21% | Patients who discontinued the study before week 12 or who received steroid injections were considered to be **non-responders** | The primary efficacy analysis assessed whether the proportion of clinical responders in the everolimus group was higher than in the placebo group and was performed with the Fisher exact test | No |
| Burmester *et al*. (2013) [4] | 399/399 | 311 | Yes | Yes | There co-primary endpoints were **ACR20** response , mean change from baseline in **HAQ-DI** and **DAS28<2.6** at week 12 | ITT | 22% | A **non-responder** imputation (NRI) was used to address missing data | Normal-approximation to difference in binomial rates was used to test for differences in rates of ACR20 response and DAS28<2.6. HAQ-DI was expressed as changes from baseline and the analysis used a mixed-effect longitudinal model | An analysis using the **LOCF** was preformed to support the robustness of the results |
|  |  |  |  |  |  |  |  |  |  |  |
|  |  |  |  |  |  |  |  |  |  |  |
|  |  |  |  |  |  |  |  |  |  |  |
|  |  |  |  |  |  |  |  |  |  |  |
|  |  |  |  |  |  |  |  |  |  |  |
|  |  |  |  |  |  |  |  |  |  |  |
|  |  |  |  |  |  |  |  |  |  |  |
|  |  |  |  |  |  |  |  |  |  |  |
| Buttgereit *et al*. (2013) [5] | 350/350 | 323 | Yes | Yes^¥^ | The primary efficacy was the proportion of patients with an **ACR20** response at week 12 | M-ITT | 8% | the primary analysis consisted in imputing all missing assessments at 3 months as **non-responder** | A logistic regression model with treatment and geographic area, age & gender as factors. | As a sensitivity analysis, the missing assessments will be imputed conditionally to the completion of the study by the patient, i.e. if a patient discontinued prematurely, the ACR20 was imputed as **non-responder**, while patients who completed the study but have a missing assessment for ACR20 at visit 4 will not be imputed. Analysis for the observed case only will be presented as a secondary sensitivity analysis. It is assumed that final efficacy assessments will be available and complete for all **PP** patients |
| Choy *et al*.  (2012) [6] | 247/243 | 163 | Yes | Yes | The primary efficacy was the **ACR20** response at week 24 | M-ITT | 34% | For ACR20 response rates, patients who withdrew before week 24 were considered to be **non-responders** from the time of withdrawal. For continuous data, missing data were imputed by **LOCF** analysis. | The ACR response rates, comparison of the treatment groups and changes from baseline in categorical variables were analysed by the CMH method stratified by country. | Sensitivity analyses, which included the use of **LOCF** imputation of missing data, were carried out for ACR20 and ACR50 response rates. ACR20 and ACR50 responses at each week also used LOCF imputation of missing data |
| Cohen *et al*. (2009) [7] | 204/204 | 166 | No | Yes | The primary efficacy  was the proportion of patients with an **ACR20** response at week 12 | ITT | 19% | Patients with missing ACR20 response values were classified as **non-responders** for the purpose of the analysis | The ACR20 response was analysed using the CMH test with region and previous MTX as stratification factors | No |
| Combe *et al*. (2009) [8] | 260/254 | 158 | No | Yes | The primary efficacy  endpoint, the percentage of patients achieving 20% improvement, defined as  **ACR20** criteria at week 24 | M-ITT | 38% | The **LOCF** approach was used to account for missing data points. **LOCF** imputation was also applied to patients who discontinued for unsatisfactory response | The ACR20, ACR50 and ACR70 response rates were analysed using the MH χ^2^ test, stratified by study centre | No |
| Dougados *et al*. (2013) [9] | 556/553 | 512 | Yes | Yes | The primary outcome was patients in **remission** according to DAS28–ESR (DAS28 <2.6) at week 24 | ITT | 7% | **Non-responder** imputation for categorical variables (e.g, DAS28 remission, ACR response), **LOCF** until patient withdrawal for missing joint counts and no additional imputation of missing values | The primary endpoint, as well as for similar endpoints, a logistic regression model was employed including the stratification factors used at randomisation (site and baseline DAS28 ≤ or >5.5) with a supportive CMH test stratified for the same parameters | No |
| Emery *et al*. (2010) [10] | 511/509 | 487 | No | Yes | The primary end point was the proportion of patients with an **ACR20** response at week 24 | ITT | 4% | Missing data, including data for patients who withdrew or received rescue treatment, were imputed using the **non-responder** method for ACR and EULAR and **LOCF** for all other end points | The efficacy of rituximab versus placebo was analysed using the CMH test for categorical end points | No |
| Emery *et al*. (2009) [11] | 637/634 | 599 | Yes | Yes | The primary end points was the difference in the **ACR50** response at week 24 between groups | ITT | 6% | ACR component data were missing were considered **non-responders** as patients meeting predefined treatment failure criteria | Treatment group differences were assessed with a 2-sided CMH test for discrete variables | No |
| Emery *et al*. (2008) [12] | 499/498 | 304 | Yes | Yes | The primary efficacy was improvement in **ACR20** response at week 24 | ITT | 39% | Patients on rescue therapy or with insufficient data to calculate the change from baseline ACR score at a specific time point were classified as **non-responders** at that time point. In the case of missing data, SJC and TJC scores were calculated as **LOCF**. There was no imputation for missing data for the remaining ACR | Primary endpoint analysis for ACR20 response compared the proportion of patients in each of the tocilizumab plus methotrexate groups versus controls with a response at week 24 using a CMH test with adjustment for site. | No |
|  |  |  |  |  |  |  |  |  |  |  |
| Emery *et al*. (2008) [13] | 542/528 | 410 | Yes | Yes | Co-primary endpoints were the proportion of patients achieving **remission** (DAS28 <2.6) at week 52 and modified **total Sharp score** from baseline to week 52 | M-ITT | 22% | Missing values were assumed to be missing at random and imputed with the **LOCF** for clinical endpoints. Patients with missing radiographs at week 52, this value was imputed by **linear extrapolation** from the time of final on-treatment assessment | The proportion of participants achieving remission as measured by DAS28 was compared with Fisher’s exact test. The change in radiographs was compared between treatment groups with ANCOVA on the rank of the change scores, averaged over readers with the rank of baseline as covariate | No |
| Fleischmann *et al*. (2012) [14] | 611/610 | 555 | Yes | No | The three primary efficacy end points were the percentage of patients with an **ACR20** response, the change from baseline **HAQ-DI** & **DAS28-ESR<2.6** at month 3 | M-ITT | 9% | Imputation of **no response** was used to account for missing data (including data from patients who withdrew from the study) in calculating these two end points (ACR20 & DAS-ESR<2.6) | The normal-approximation test for the difference in binomial proportions was used to determine the superiority of treatment versus placebo with respect to two of the primary endpoints. The changes in HAQ-DI was analysed a mixed effect longitudinal model that included effects of treatment and visit. | No |
| Fleischmann *et al*. (2009) [15] | 220/220 | 104 | Yes | Yes | The primary efficacy endpoint was **ACR20** response at  week 24 | M-ITT | 53% | The actual number of subjects in the summaries varies slightly from the M-ITT numbers due to non-imputable missing data for each parameter. Patients who withdrew for any reason were considered **non-responders.** | The proportion of ACR20 responders/non-responders  at each visit was compared using the CMH test stratified by country | Several sensitivity analyses of the primary efficacy variable were conducted, including **LOCF** analysis |
|  |  |  |  |  |  |  |  |  |  |  |
|  |  |  |  |  |  |  |  |  |  |  |
|  |  |  |  |  |  |  |  |  |  |  |
|  |  |  |  |  |  |  |  |  |  |  |
|  |  |  |  |  |  |  |  |  |  |  |
|  |  |  |  |  |  |  |  |  |  |  |
| Gabay *et al*. (2013) [16] | 326/325 | 257 | Yes | Yes | The primary efficacy endpoint was change in **DAS28** from baseline to week 24 | ITT | 21% | We imputed missing data by carrying the **LOCF** | We assessed the primary endpoint by an ANCOVA  model that included treatment group, stratification factors, and baseline score | To assess the sensitivity of the primary efficacy endpoint, analysis of covariance (ANCOVA) was repeated using the **PP** and another using observed data (no imputation was performed for missing DAS28 components at week 24, thus excluding patients who withdrew or received escape therapy. Further, sensitivity analysis using the missing at random data mechanism was conducted |
| Genovese *et al*. (2011) [17] | 302/301 | 227 | Yes | No | The primary efficacy was improvement in **ACR20** response at week 12 | ITT | 25% | ACR responses were calculated using **a non-responder** imputation. Any patients who received prohibited concomitant therapies were considered **non-responders**. **LOCF** imputation was used to handle missing values up to week 12 | Differences in the proportion of ACR20 responders between groups were evaluated using Dunnett’s modification for the chi-squared test | No |
| Genovese *et al.*(2011) [18] | 1457/1457 | 1369 | Yes | Yes | The primary efficacy was improvement in **ACR20** response at month 6 | ITT & PP | 6% | Patients who discontinued were considered **non responders**. For mean change in HAQ DI and DAS28-CRP, missing values were imputed using a **LOCF** (we excluded patients for whom only baseline observations were available) | Treatment differences  were calculated for efficacy assessments, with 95% Cis for ACR or HAQ-DI responses | No |
| Genovese *et al*. (2008) [19] | 1,220/1,216 | 1,056 | Yes | Yes | The primary efficacy was improvement in **ACR20 response** at week 24 | ITT | 13% | Patients who did not have required data at specific time point, who withdrew from the study or who received rescue therapy were classified as **non-responders** | ACR20 response was compared using a CMH χ^2^ test with adjustment for site | No |
| Jones *et al*.  (2010) [20] | 673/673 | 583 | Yes | Yes | The primary end point was the proportion of patients with an **ACR20** response at week 24 | ITT & PP | 13% | Not stated | ACR20 response rates were analysed using extended CMH, with adjustment stratification (site and disease duration) | No |
| Kavanaugh *et al*. (2013) [21] | 1,032/1,032 |  | Yes | No | The primary endpoint was a composite of **DAS28 (CRP) <** 3.2 at week 78 but evaluated at week 26 | ITT & observed analyses | 10% | For categorical clinical and functional outcomes, a **non-responder** imputation approach was used, such that patients with missing responses were considered non-responders. **LOCF** analyses were used for continuous clinical and functional outcomes. **MI** was used to assess radiographic data | Categorical efficacy variables were analysed using Pearson’s χ2 test or Fisher’s exact test, evaluations of continuous variables were based on an analysis of covariance model adjusting for baseline. Regression analyses examined variables associated with the stable (DAS28 (CRP) <3.2 at both weeks 22 and 26 | No |
| Keystone *et al*. (2008) [22] | 520/499 | 374 | No | No | Primary end point was the proportion of patients with an **ACR20** response at week 24 | ITT | 25% | **LOCF** method was used to replace any missing values; therefore, all patients except those with missing baseline values were included in the analysis. | We created 2 x 2 tables to examine the frequency of discrepant responses on ACR20 scores | No |
| Keystone *et al*. (2008) [23] | 982/982 | 572 | No | Yes | Co-Primary-end points were the response according to the **ACR20** criteria at week 24 and mean change of **total Sharp score** at week 52 | ITT | 42% | Primary analyses were performed using **non-responder** imputation. the modified total Sharp score at week 52 was estimated by **linear extrapolation** | Logistic regression with treatment and geographic region as factors was used to compare active treatment and placebo in ACR responses. ANCOVA was used to analyse the total Sharp score with region as a factor and with ranked baseline total Sharp score as covariate | Multiple sensitivity analyses were performed on the radiographic data under various assumptions on the imputation of missing values, including an analysis of the **PP** population, which consisted of a subset of the **ITT** population, excluding patients who had at least 1 major protocol deviation, as confirmed during a pre-analysis review prior to unblinding of the data. Sensitivity analyses were also performed using the **LOCF** method for imputation of missing scores |
|  |  |  |  |  |  |  |  |  |  |  |
| Kremer *et al*. (2010) [24] | 643/643 | 566 | Yes | Yes | The primary end point was the proportion of patients achieving an **ACR50** response at week 14 | ITT | 12% | A **LOCF** procedure was used to impute missing ACR response data if the patient had data for at least 1 measured ACR response component at week 14 | Differences between treatment groups were assessed with CMH test for discrete variables | No |
| Lu *et al*. (2009) [25] | 492/489 | 433 | No | Yes^¥^ | Primary-end points was the proportion of patients with improvement in **ACR20** response at week 24 | ITT | 11% | Analyses used the **LOCF** method. | Overall assessments were compared by means of the CMH test | No |
| Machold *et al*. (2010) [26] | 389/383 | 303 | Yes | Yes^¥^ | The primary end point was defined as the drug free clinical **remission** both at week 12 and 52 | ITT | 21% | When patients were lost to follow-up, treatment was considered to have been a failure from that time point onward. The data of these patients were analysed using the last observation before DMARD/GC treatment or drop-out carried forward ( **LOCF**) | Categorical variables were analysed using Fisher’s exact or χ2 test | For an additional sensitivity analysis, we assumed that all patients who were lost to follow-up and did not receive DMARDs up to their last visit went into remission ( rather than assuming **non-response** as is usually done in clinical trial settings) |
|  |  |  |  |  |  |  |  |  |  |  |
| Mease *et al*. (2010) [27] | 475/475 | 425 | Yes | Yes^¥^ | The primary endpoint was improvement in **ACR20** at week 48 | ITT | 11% | **LOCF** method was used to impute missing components. | The difference in ACR20 response rates between the placebo retreatment group and the rituximab treatment group was tested using the CMH test statistic, stratified according to baseline rheumatoid factor status and ≥ 20% improvement in both SJC and TJC at Week 24 | No |
| Moreland *et al*. (2012) [28] | 755/755 | 476 | Yes | Yes^¥^ | The primary end point for the trial was an observed-group analysis of the **DAS28-ESR** between week 48 and week 102 | ITT | 37% | Results were presented as all observed data without imputation for missing values, or as a **non-responders** analysis as indicated. | The primary outcome of the study (DAS28-ESR ) was analysed using a two-way, repeated-measures, mixed-models analysis | We performed the analysis using several different missing-data approaches, and regardless of the method used, there were no differences in the results |
| Pavelka *et al*. (2009) [29] | 141/140 | 115 | Yes | Yes | The primary study endpoint was a change in the **DAS28** after 28 weeks | ITT | 18% | The last observation carried forward (**LOCF**) were used | Analysis of covariance (ANCOVA) was used to examine statistical differences between groups adjusted for the baseline values | No |
| Rigby *et al*. (2012) [30] | 1015/1006 | 806 | Yes | Yes | The primary endpoint was improvement in **ACR20** response at weeks 24 and 48 | ITT | 20% | Patients who withdrew early or those who received rescue medication were considered **non-responders** | ACR20 response rate between the groups was analysed the CMH test statistic, stratifying by region and baseline RF status | No |
| Rubbert-Roth *et al*. (2010) [31] | 378/377 | 314 | Yes | Yes | The primary endpoint was the proportion of patient with an **ACR20** response at week 48 | ITT | 18% | Missing data were imputed using the **non-responder** method for ACR and EULAR **LOCF** was used for all other endpoints. | For the primary efficacy variable ACR20 response at week 48, the treatment group was compared with CMH test and logistic regression analysis, adjusted for baseline factors of Rheumatoid Factor status, region and earlier biological use. | No |
|  |  |  |  |  |  |  |  |  |  |  |
|  |  |  |  |  |  |  |  |  |  |  |
| Schiff *et al*. (2008) [32] | 431/431 | 406 | Yes | Yes | The primary study endpoint was **DAS28-ESR** at 6 months | ITT | 6% | Patients who discontinued the study prematurely were considered as **non-responders** subsequent to the time of discontinuation for ACR responses, good EULAR responses and clinically meaningful HAQ-DI responses. For all continuous measurements (mean changes in DAS28 and the HAQ-DI score) the last observations prior to the discontinuation were carried forward (**LOCF**) | Treatment group was compared by analyses of covariance (ANCOVA) for mean changes from baseline in DAS28 (ESR). | No |
| Smolen *et al*. (2013) [33] | 604/597 | 497 | Yes | Yes | The primary endpoint was the proportion of patients with **low disease activity** (DAS28-ESR) ≤3.2 at week 88 | M-ITT | 17% | Patients who discontinued early because of poor efficacy were imputed **as non-responders** for all time points. All other patients were analysed with the **LOCF** method | The primary endpoint was analysed with the CMH test of general association | sensitivity analyses of the proportions of patients with DAS28 (low disease activity ) at week 88, with NRI, LOCF, PP & completers was conducted |
| Smolen *et al*. (2009) [34] | 461/459 | 295 | Yes | Yes | The primary endpoint was improvement in **ACR20** response at week 14 | ITT | 36% | Patients who were missing all components of the ACR or DAS response criteria were regarded as **non- responders** | The primary endpoint was analysed the CMH test stratified by baseline methotrexate use | No |
| Smolen *et al*. (2009) [35] | 619/619 | 372 | Yes | Yes | The primary end point was **ACR20** response at week 24 | ITT | 40% | Primary analysis used **non-responder** imputation. The number of subjects in the summaries varies slightly from the ITT numbers owing to non-imputable missing data. Individual ACR Core set components were imputed by **LOCF** | Treatment comparisons between the groups were performed using logistic regression with factors for treatment and region. Individual ACR Core set components were analysed using analysis of covariance, with region and treatment group as factors and baseline value as covariate. | Sensitivity analyses were performed under various assumptions on the imputation of missing values, including **LOCF** imputation of missing scores. |
|  |  |  |  |  |  |  |  |  |  |  |
| Smolen *et al*. (2008) [36] | 623/622 | 455 | Yes | Yes^¥^ | The primary endpoint was the proportion of patients with improvement in **ACR20** response at week 24 | ITT | 27% | Patients who withdrew before week 24, patients who received rescue therapy, and patients whose week 24 categorical endpoints could not be determined due to insufficient data were deemed to be **non-responders** in the analysis. **LOCF** was used for tender and swollen joint counts. No imputation was used for missing HAQ, CRP, ESR and Global VAS data | ACR20 response was compared between treatment groups with a CMH and χ2 test with adjustment for site | No |
|  |  |  |  |  |  |  |  |  |  |  |
| Tak *et al*.  (2012) [37] | 840/836 | 590 | Yes | Yes | Efficacy end points were the proportion of patients with **ACR20** response at weeks 24 and 48 | ITT | 29% | Patients with missing data and those who received rescue medications were considered **non-responders** | ACR20 response rate between treatment group was analysed the CMH test, stratifying by region and baseline DMARD therapy (MTX/LEF) | No |
| Tylor *et al*.  (2011) [38] | 265/260 | 238 | Yes | Yes | The primary endpoint was the **ACR20** response at week 24 | ITT | 8% | For categorical endpoints, withdrew from the study were imputed as **non-responders**. For continuous endpoints, data were imputed by **LOCF** before taking disallowed medication or withdrawal | For categorical endpoints, the efficacy of ofatumumab versus  placebo was analysed using the CMH test, adjusting for baseline stratification factors, RF status and region | No |
| Urata *et al*.  (2012) [39] | 243/243 | 222 | Yes | No | Primary outcome measures consisted of the proportion of patients in clinical **remission** (DAS28 <2.6) at 56 weeks | ITT | 9% | Patients who had to withdraw  before completing 56 weeks of the study were designated as **non-responders** at 56 weeks | Outcomes for patients between treatment groups were compared using Student’s t test, the Mann–Whitney U test, Kruskal–Wallis and the χ 2 test, as appropriate | No |
| van der Heijde *et al*.(2013)[40] | 800/797 | 643 | Yes | Yes | Co-primary efficacy end points evaluated with respect to the response rates according to the **ACR20** response rates and total **Sharp score** at month 6 | ITT | 19% | ACR20 response rate and DAS28-ESR <2.6; **non-responder** imputation (NRI; setting the ACR20 response rate or the rate of DAS28-ESR <2.6 to nonresponsive) addressed missing data. Patients who advanced before month 6 (non-responders) had their month 6 measurements imputed using a **linear extrapolation** from month 3 radiographs even when month 6 radiographs were available, regardless of treatment  Assignment | Associated binary variables (e.g., rates of patients with no progression) were analysed using normal approximation to the binomial. the primary analysis was an analysis of variance model for change from baseline to month 6, and included baseline total SHS as a covariate | Sensitivity analyses, including multiple imputation/generalized estimating equation analyses for ACR and 4-variable DAS28-ESR<2.6 response rates |
| van Vollenhoven *et al*. (2012) [41] | 717/717 | 556 | Yes | Yes | The three primary efficacy end points were the percentage of patients with an **ACR20** response & **DAS28-ESR<2.6** at month 6; the mean change in **HAQ-DI** at month 3 | ITT | 22% | Imputation of **no-response** was used to account for missing data in the calculation in ACR and DAS28-ESR at month 6. However, in this trial, the imputation of no response was also applied to patients who did not have a 20% reduction in the number of tender and swollen joints at month 3, regardless of treatment assignment. | The normal approximation for the difference in binomial proportions was used to test the superiority of each of the two tofacitinib regimens over placebo with respect to ACR20 response rates and DAS28-ESR<2.6 | No |
| van Vollenhoven *et al*. (2009) [42] | 258/258 | 194 | Yes | Yes | Primary outcome was achievement of a good response according to European League Against Rheumatism (**EULAR**) at month 12 | ITT | 25% | We used **non-responder** imputation for patients who, during first 12 months of the trial could not or did not continue treatment as per protocol for any reason. | We used Fisher’s exact test to compare the proportion with EULAR good response or ACR20. We used the χ2 test to compare nominal disease-activity categories | No |
| Wei *et al*.  (2009) [43] | 503/503 | 454 | No | No | The primary efficacy variable was the **ACR** definition of **20%** improvement in RA at week 24 | ITT | 10% | Not stated | Chi-square and Fisher exact test was used for categorical variables, and *t* analysis of variance was used for continuous variables | No |
|  |  |  |  |  |  |  |  |  |  |  |
|  |  |  |  |  |  |  |  |  |  |  |
|  |  |  |  |  |  |  |  |  |  |  |
|  |  |  |  |  |  |  |  |  |  |  |
| Weinblatt *et al*. (2013) [44] | 592/592 | 502 | Yes | Yes | The primary endpoint was the proportion of patients with an **ACR20** response at week 14. | ITT | 15% | Patients who discontinued because of lack of efficacy before week 14 and who lacked all week 14 ACR20 component data for any reason were considered ACR20 **non-responders** at week 14 through week 24. **LOCF** procedure was employed to impute missing ACR component data (e.g. swollen or tender joint count, or global assessments of disease) at week 14 if the patient had data for at least one other ACR component at week 14 | Proportions of patients achieving response levels were compared between treatment groups using a two-sided CMH test stratified by screening CRP (<1.5 mg/dl, ≥1.5 mg/dl) | Sensitivity analyses of the primary endpoint included analyses in which patients who: (1) discontinued study agent due to an AE, (2) had insufficient data to determine ACR20 response and were considered ‘non-responders, and (3) whose treatment regimen was inadvertently unblinded during the study were excluded. A fourth sensitivity analysis utilised a re-randomisation test |
| Weinblatt *et al*. (2013) [45] | 646/646 | 543 | Yes | Yes | The primary outcome measure was the proportion of patients  achieving response on the **ACR20** improvement at 1 year | ITT &PP | 16% | All patients who prematurely discontinued the study after receiving the study drug, regardless of the reason, were considered **non-responders** at all scheduled visits subsequent to the point of discontinuation. | For the response rates determined in efficacy analyses, the estimate of the proportion of responders (with 95% CIs) within each treatment group, as well as the difference in response rates between treatment groups, are presented | No |
| Weinblatt *et al*. (2012) [46] | 1,063/1,063 | 955 | Yes | Yes | The primary efficacy end point was the **ACR20** response rate at week 12 | ITT &PP | 10% | Primary efficacy analysis used **non-responder** imputation (e.g. patients with missing ACR20 response data at week 12, for any reason, were designated **non-responders**). For continuous data, missing data were imputed by **LOCF** analysis (excluding patients for whom only baseline observations were available). | Treatment comparisons were performed using logistic regression with factors for treatment, concomitant use of MTX at baseline, previous TNF inhibitor use and disease duration . | No |
| Weinblatt *et al*. (2008) [47] | 189/189 | 158 | Yes | Yes | The primary efficacy end point was the **ACR20** response rate at week 12 | ITT | 16% | Any patient who withdrew from the study prior to week 12 for any reason was defined as a **non-responder** | Efficacy data (ACR20,50 and 70) at each post-dosing visit) were compared between each treatment group and the placebo group using Pearson’s chi-square test | No |
|  |  |  |  |  |  |  |  |  |  |  |
| Weinblatt *et al*. (2008) [48] | 201/200 | 187 | Yes | Yes | The primary end point of the study was the proportion of patients with a good or moderate defined by **EULAR** criteria at week 12 | ITT | 7% | **Non-responder** imputation was used for categorical responses such as the EULAR response and DAS28 remission, and the **LOCF** method was used for continuous variables, such as the percentage improvement in the DAS28 score | Differences in the various end points at week 12 between those taking etanercept 50 mg twice weekly and those taking etanercept 50 mg once weekly were tested | Additional supportive analyses were performed on the subset of patients who were evaluated at specific visits (e.g. completers) |
| Westhovens *et al*. (2009) [49] | 509/509 | 459 | Yes | Yes¥ | Co-primary, r**emission** at year 1, defined as a disease activity score in 28 joints (DAS28- CRP) of less than 2.6 and structural damage at year 1 | ITT | 10% | For DAS28 (CRP)-defined remission, ACR and HAQ-DI responses; patients who discontinued were considered **non-responders** subsequent to discontinuation. For mean change from baseline in DAS28 (CRP), a **LOCF** imputation was applied. **Linear extrapolation** for patients with radiographs at baseline and either month 6 or discontinuation (or both) | For DAS28 (CRP)-defined remission responder rates, ACR at year 1, a continuity-corrected x^2^ test was used. For change from baseline for DAS28 (CRP), comparisons were based on an analysis of covariance model, including treatment as the main factor and baseline value as a covariate. A non-parametric analysis of covariance model was used for change from baseline to year 1 structural damage | For DAS28 (CRP)-defined remission and the proportion of patients without radiographic progression, additional analyses were performed in which all patients who received additional non-biological DMARD and/or a steroid pulse were considered non-responders. For mean change from baseline to year 1 in TS an analysis was performed considering only completers |
| Yazici *et al*. (2012) [50] | 619/614 | 409 | Yes | No | The primary efficacy endpoint was the **ACR50** response at week 24 | ITT | 33% | Patients who received rescue therapy and patients who did not have data required to assess efficacy outcomes at week 24 were classified as **non-responders**. **LOCF** methodology was used for missing joint count data. | Fisher’s exact test was used to determine p values for ACR responses, and DAS28 remission differences between groups at each time point. | No |
| Zhang *et al*. (2008) [51] | 236/236 | 211 | Yes | No | The primary efficacy variable was the **ACR20** response at week 24 | ITT & completers | 11% | Not stated | Efficacy analysis of outcome variables was based on mean changes from baseline to end point in the ITT population. Categorical variables were analysed by chi-square test with Fisher’s exact test and continuous variables were analysed by *t*-test and analysis of variance | No |

^¥^reported percentage of anticipated dropout rate in power calculation; CHM= Cochran Mantel Haenszel; MH= Mantel Haenszel; ACR=American College of Rheumatology; DAS28= Disease Activity Score for 28 joints; EULAR=European League of Associations for Rheumatology; TJ= Tender Joint count; SJ= Swollen Joint count; mTSS= Modified Total Sharp Score (radiography); VAS= Visual analogue scale; ESR=Erthrocyte Sedimentation rate; CRP=C-reactive Protein; HAQ or HAQ-DI=Health Assessment Questionnaire; LOCF=Last observation carried forward; MI=multiple imputation; ITT= intention-to-treat; PP= per protocol, M-ITT=Modified intention-to-treat

# References

1. Alten RE, Zerbini C, Jeka S, Irazoque F, Khatib F, Emery P et al. Efficacy and safety of pamapimod in patients with active rheumatoid arthritis receiving stable methotrexate therapy. Ann Rheum Dis. 2010;69(2):364-7. doi:10.1136/ard.2008.104802.

2. Braun J, Kastner P, Flaxenberg P, Wahrisch J, Hanke P, Demary W et al. Comparison of the clinical efficacy and safety of subcutaneous versus oral administration of methotrexate in patients with active rheumatoid arthritis: results of a six-month, multicenter, randomized, double-blind, controlled, phase IV trial. Arthritis Rheum. 2008;58(1):73-81. doi:10.1002/art.23144.

3. Bruyn GA, Tate G, Caeiro F, Maldonado-Cocco J, Westhovens R, Tannenbaum H et al. Everolimus in patients with rheumatoid arthritis receiving concomitant methotrexate: a 3-month, double-blind, randomised, placebo-controlled, parallel-group, proof-of-concept study. Ann Rheum Dis. 2008;67(8):1090-5. doi:10.1136/ard.2007.078808.

4. Burmester GR, Blanco R, Charles-Schoeman C, Wollenhaupt J, Zerbini C, Benda B et al. Tofacitinib (CP-690,550) in combination with methotrexate in patients with active rheumatoid arthritis with an inadequate response to tumour necrosis factor inhibitors: a randomised phase 3 trial. Lancet. 2013;381(9865):451-60. doi:10.1016/s0140-6736(12)61424-x.

5. Buttgereit F, Mehta D, Kirwan J, Szechinski J, Boers M, Alten RE et al. Low-dose prednisone chronotherapy for rheumatoid arthritis: a randomised clinical trial (CAPRA-2). Ann Rheum Dis. 2013;72(2):204-10. doi:10.1136/annrheumdis-2011-201067.

6. Choy E, McKenna F, Vencovsky J, Valente R, Goel N, Vanlunen B et al. Certolizumab pegol plus MTX administered every 4 weeks is effective in patients with RA who are partial responders to MTX. Rheumatology (Oxford). 2012;51(7):1226-34. doi:10.1093/rheumatology/ker519.

7. Cohen SB, Cheng TT, Chindalore V, Damjanov N, Burgos-Vargas R, Delora P et al. Evaluation of the efficacy and safety of pamapimod, a p38 MAP kinase inhibitor, in a double-blind, methotrexate-controlled study of patients with active rheumatoid arthritis. Arthritis Rheum. 2009;60(2):335-44. doi:10.1002/art.24266.

8. Combe B, Codreanu C, Fiocco U, Gaubitz M, Geusens PP, Kvien TK et al. Efficacy, safety and patient-reported outcomes of combination etanercept and sulfasalazine versus etanercept alone in patients with rheumatoid arthritis: a double-blind randomised 2-year study. Ann Rheum Dis. 2009;68(7):1146-52. doi:10.1136/ard.2007.087106.

9. Dougados M, Kissel K, Sheeran T, Tak PP, Conaghan PG, Mola EM et al. Adding tocilizumab or switching to tocilizumab monotherapy in methotrexate inadequate responders: 24-week symptomatic and structural results of a 2-year randomised controlled strategy trial in rheumatoid arthritis (ACT-RAY). Ann Rheum Dis. 2013;72(1):43-50. doi:10.1136/annrheumdis-2011-201282.

10. Emery P, Deodhar A, Rigby WF, Isaacs JD, Combe B, Racewicz AJ et al. Efficacy and safety of different doses and retreatment of rituximab: a randomised, placebo-controlled trial in patients who are biological naive with active rheumatoid arthritis and an inadequate response to methotrexate (Study Evaluating Rituximab's Efficacy in MTX iNadequate rEsponders (SERENE)). Ann Rheum Dis. 2010;69(9):1629-35. doi:10.1136/ard.2009.119933.

11. Emery P, Fleischmann RM, Moreland LW, Hsia EC, Strusberg I, Durez P et al. Golimumab, a human anti-tumor necrosis factor alpha monoclonal antibody, injected subcutaneously every four weeks in methotrexate-naive patients with active rheumatoid arthritis: twenty-four-week results of a phase III, multicenter, randomized, double-blind, placebo-controlled study of golimumab before methotrexate as first-line therapy for early-onset rheumatoid arthritis. Arthritis Rheum. 2009;60(8):2272-83. doi:10.1002/art.24638.

12. Emery P, Keystone E, Tony HP, Cantagrel A, van Vollenhoven R, Sanchez A et al. IL-6 receptor inhibition with tocilizumab improves treatment outcomes in patients with rheumatoid arthritis refractory to anti-tumour necrosis factor biologicals: results from a 24-week multicentre randomised placebo-controlled trial. Ann Rheum Dis. 2008;67(11):1516-23. doi:10.1136/ard.2008.092932.

13. Emery P, Breedveld FC, Hall S, Durez P, Chang DJ, Robertson D et al. Comparison of methotrexate monotherapy with a combination of methotrexate and etanercept in active, early, moderate to severe rheumatoid arthritis (COMET): a randomised, double-blind, parallel treatment trial. Lancet. 2008;372(9636):375-82. doi:10.1016/s0140-6736(08)61000-4.

14. Fleischmann R, Kremer J, Cush J, Schulze-Koops H, Connell CA, Bradley JD et al. Placebo-controlled trial of tofacitinib monotherapy in rheumatoid arthritis. N Engl J Med. 2012;367(6):495-507. doi:10.1056/NEJMoa1109071.

15. Fleischmann R, Vencovsky J, van Vollenhoven RF, Borenstein D, Box J, Coteur G et al. Efficacy and safety of certolizumab pegol monotherapy every 4 weeks in patients with rheumatoid arthritis failing previous disease-modifying antirheumatic therapy: the FAST4WARD study. Ann Rheum Dis. 2009;68(6):805-11. doi:10.1136/ard.2008.099291.

16. Gabay C, Emery P, van Vollenhoven R, Dikranian A, Alten R, Pavelka K et al. Tocilizumab monotherapy versus adalimumab monotherapy for treatment of rheumatoid arthritis (ADACTA): a randomised, double-blind, controlled phase 4 trial. The Lancet. 2013;381(9877):1541-50.

17. Genovese MC, Cohen SB, Wofsy D, Weinblatt ME, Firestein GS, Brahn E et al. A 24-week, randomized, double-blind, placebo-controlled, parallel group study of the efficacy of oral SCIO-469, a p38 mitogen-activated protein kinase inhibitor, in patients with active rheumatoid arthritis. J Rheumatol. 2011;38(5):846-54. doi:10.3899/jrheum.100602.

18. Genovese MC, Covarrubias A, Leon G, Mysler E, Keiserman M, Valente R et al. Subcutaneous abatacept versus intravenous abatacept: a phase IIIb noninferiority study in patients with an inadequate response to methotrexate. Arthritis Rheum. 2011;63(10):2854-64. doi:10.1002/art.30463.

19. Genovese MC, McKay JD, Nasonov EL, Mysler EF, da Silva NA, Alecock E et al. Interleukin-6 receptor inhibition with tocilizumab reduces disease activity in rheumatoid arthritis with inadequate response to disease-modifying antirheumatic drugs: the tocilizumab in combination with traditional disease-modifying antirheumatic drug therapy study. Arthritis Rheum. 2008;58(10):2968-80. doi:10.1002/art.23940.

20. Jones G, Sebba A, Gu J, Lowenstein MB, Calvo A, Gomez-Reino JJ et al. Comparison of tocilizumab monotherapy versus methotrexate monotherapy in patients with moderate to severe rheumatoid arthritis: the AMBITION study. Ann Rheum Dis. 2010;69(1):88-96. doi:10.1136/ard.2008.105197.

21. Kavanaugh A, Fleischmann RM, Emery P, Kupper H, Redden L, Guerette B et al. Clinical, functional and radiographic consequences of achieving stable low disease activity and remission with adalimumab plus methotrexate or methotrexate alone in early rheumatoid arthritis: 26-week results from the randomised, controlled OPTIMA study. Ann Rheum Dis. 2013;72(1):64-71. doi:10.1136/annrheumdis-2011-201247.

22. Keystone E, Burmester GR, Furie R, Loveless JE, Emery P, Kremer J et al. Improvement in patient-reported outcomes in a rituximab trial in patients with severe rheumatoid arthritis refractory to anti-tumor necrosis factor therapy. Arthritis Rheum. 2008;59(6):785-93. doi:10.1002/art.23715.

23. Keystone E, Heijde D, Mason D, Jr., Landewe R, Vollenhoven RV, Combe B et al. Certolizumab pegol plus methotrexate is significantly more effective than placebo plus methotrexate in active rheumatoid arthritis: findings of a fifty-two-week, phase III, multicenter, randomized, double-blind, placebo-controlled, parallel-group study. Arthritis Rheum. 2008;58(11):3319-29. doi:10.1002/art.23964.

24. Kremer J, Ritchlin C, Mendelsohn A, Baker D, Kim L, Xu Z et al. Golimumab, a new human anti-tumor necrosis factor alpha antibody, administered intravenously in patients with active rheumatoid arthritis: Forty-eight-week efficacy and safety results of a phase III randomized, double-blind, placebo-controlled study. Arthritis Rheum. 2010;62(4):917-28. doi:10.1002/art.27348.

25. Lu LJ, Bao CD, Dai M, Teng JL, Fan W, Du F et al. Multicenter, randomized, double-blind, controlled trial of treatment of active rheumatoid arthritis with T-614 compared with methotrexate. Arthritis Rheum. 2009;61(7):979-87. doi:10.1002/art.24643.

26. Machold KP, Landewe R, Smolen JS, Stamm TA, van der Heijde DM, Verpoort KN et al. The Stop Arthritis Very Early (SAVE) trial, an international multicentre, randomised, double-blind, placebo-controlled trial on glucocorticoids in very early arthritis. Ann Rheum Dis. 2010;69(3):495-502. doi:10.1136/ard.2009.122473.

27. Mease PJ, Cohen S, Gaylis NB, Chubick A, Kaell AT, Greenwald M et al. Efficacy and safety of retreatment in patients with rheumatoid arthritis with previous inadequate response to tumor necrosis factor inhibitors: results from the SUNRISE trial. J Rheumatol. 2010;37(5):917-27. doi:10.3899/jrheum.090442.

28. Moreland LW, O'Dell JR, Paulus HE, Curtis JR, Bathon JM, St Clair EW et al. A randomized comparative effectiveness study of oral triple therapy versus etanercept plus methotrexate in early aggressive rheumatoid arthritis: the treatment of Early Aggressive Rheumatoid Arthritis Trial. Arthritis Rheum. 2012;64(9):2824-35. doi:10.1002/art.34498.

29. Pavelka K, Jarosova K, Suchy D, Senolt L, Chroust K, Dusek L et al. Increasing the infliximab dose in rheumatoid arthritis patients: a randomised, double blind study failed to confirm its efficacy. Ann Rheum Dis. 2009;68(8):1285-9. doi:10.1136/ard.2008.090860.

30. Rigby W, Tony HP, Oelke K, Combe B, Laster A, von Muhlen CA et al. Safety and efficacy of ocrelizumab in patients with rheumatoid arthritis and an inadequate response to methotrexate: results of a forty-eight-week randomized, double-blind, placebo-controlled, parallel-group phase III trial. Arthritis Rheum. 2012;64(2):350-9. doi:10.1002/art.33317.

31. Rubbert-Roth A, Tak PP, Zerbini C, Tremblay JL, Carreno L, Armstrong G et al. Efficacy and safety of various repeat treatment dosing regimens of rituximab in patients with active rheumatoid arthritis: results of a Phase III randomized study (MIRROR). Rheumatology (Oxford). 2010;49(9):1683-93. doi:10.1093/rheumatology/keq116.

32. Schiff M, Keiserman M, Codding C, Songcharoen S, Berman A, Nayiager S et al. Efficacy and safety of abatacept or infliximab vs placebo in ATTEST: a phase III, multi-centre, randomised, double-blind, placebo-controlled study in patients with rheumatoid arthritis and an inadequate response to methotrexate. Ann Rheum Dis. 2008;67(8):1096-103. doi:10.1136/ard.2007.080002.

33. Smolen JS, Nash P, Durez P, Hall S, Ilivanova E, Irazoque-Palazuelos F et al. Maintenance, reduction, or withdrawal of etanercept after treatment with etanercept and methotrexate in patients with moderate rheumatoid arthritis (PRESERVE): a randomised controlled trial. Lancet. 2013;381(9870):918-29. doi:10.1016/s0140-6736(12)61811-x.

34. Smolen JS, Kay J, Doyle MK, Landewe R, Matteson EL, Wollenhaupt J et al. Golimumab in patients with active rheumatoid arthritis after treatment with tumour necrosis factor alpha inhibitors (GO-AFTER study): a multicentre, randomised, double-blind, placebo-controlled, phase III trial. Lancet. 2009;374(9685):210-21. doi:10.1016/s0140-6736(09)60506-7.

35. Smolen J, Landewe RB, Mease P, Brzezicki J, Mason D, Luijtens K et al. Efficacy and safety of certolizumab pegol plus methotrexate in active rheumatoid arthritis: the RAPID 2 study. A randomised controlled trial. Ann Rheum Dis. 2009;68(6):797-804. doi:10.1136/ard.2008.101659.

36. Smolen JS, Beaulieu A, Rubbert-Roth A, Ramos-Remus C, Rovensky J, Alecock E et al. Effect of interleukin-6 receptor inhibition with tocilizumab in patients with rheumatoid arthritis (OPTION study): a double-blind, placebo-controlled, randomised trial. Lancet. 2008;371(9617):987-97. doi:10.1016/s0140-6736(08)60453-5.

37. Tak PP, Mease PJ, Genovese MC, Kremer J, Haraoui B, Tanaka Y et al. Safety and efficacy of ocrelizumab in patients with rheumatoid arthritis and an inadequate response to at least one tumor necrosis factor inhibitor: results of a forty-eight-week randomized, double-blind, placebo-controlled, parallel-group phase III trial. Arthritis Rheum. 2012;64(2):360-70.

38. Taylor PC, Quattrocchi E, Mallett S, Kurrasch R, Petersen J, Chang DJ. Ofatumumab, a fully human anti-CD20 monoclonal antibody, in biological-naive, rheumatoid arthritis patients with an inadequate response to methotrexate: a randomised, double-blind, placebo-controlled clinical trial. Ann Rheum Dis. 2011;70(12):2119-25. doi:10.1136/ard.2011.151522.

39. Urata Y, Uesato R, Tanaka D, Nakamura Y, Motomura S. Treating to target matrix metalloproteinase 3 normalisation together with disease activity score below 2.6 yields better effects than each alone in rheumatoid arthritis patients: T-4 Study. Ann Rheum Dis. 2012;71(4):534-40. doi:10.1136/annrheumdis-2011-200108.

40. van der Heijde D, Tanaka Y, Fleischmann R, Keystone E, Kremer J, Zerbini C et al. Tofacitinib (CP-690,550) in patients with rheumatoid arthritis receiving methotrexate: twelve-month data from a twenty-four-month phase III randomized radiographic study. Arthritis Rheum. 2013;65(3):559-70. doi:10.1002/art.37816.

41. van Vollenhoven RF, Fleischmann R, Cohen S, Lee EB, Garcia Meijide JA, Wagner S et al. Tofacitinib or adalimumab versus placebo in rheumatoid arthritis. N Engl J Med. 2012;367(6):508-19. doi:10.1056/NEJMoa1112072.

42. van Vollenhoven RF, Ernestam S, Geborek P, Petersson IF, Coster L, Waltbrand E et al. Addition of infliximab compared with addition of sulfasalazine and hydroxychloroquine to methotrexate in patients with early rheumatoid arthritis (Swefot trial): 1-year results of a randomised trial. Lancet. 2009;374(9688):459-66. doi:10.1016/s0140-6736(09)60944-2.

43. Wei W, Zhang LL, Xu JH, Xiao F, Bao CD, Ni LQ et al. A multicenter, double-blind, randomized, controlled phase III clinical trial of chicken type II collagen in rheumatoid arthritis. Arthritis Res Ther. 2009;11(6):R180. doi:10.1186/ar2870.

44. Weinblatt ME, Bingham CO, 3rd, Mendelsohn AM, Kim L, Mack M, Lu J et al. Intravenous golimumab is effective in patients with active rheumatoid arthritis despite methotrexate therapy with responses as early as week 2: results of the phase 3, randomised, multicentre, double-blind, placebo-controlled GO-FURTHER trial. Ann Rheum Dis. 2013;72(3):381-9. doi:10.1136/annrheumdis-2012-201411.

45. Weinblatt ME, Schiff M, Valente R, van der Heijde D, Citera G, Zhao C et al. Head-to-head comparison of subcutaneous abatacept versus adalimumab for rheumatoid arthritis: findings of a phase IIIb, multinational, prospective, randomized study. Arthritis Rheum. 2013;65(1):28-38. doi:10.1002/art.37711.

46. Weinblatt ME, Fleischmann R, Huizinga TW, Emery P, Pope J, Massarotti EM et al. Efficacy and safety of certolizumab pegol in a broad population of patients with active rheumatoid arthritis: results from the REALISTIC phase IIIb study. Rheumatology (Oxford). 2012;51(12):2204-14. doi:10.1093/rheumatology/kes150.

47. Weinblatt ME, Kavanaugh A, Burgos-Vargas R, Dikranian AH, Medrano-Ramirez G, Morales-Torres JL et al. Treatment of rheumatoid arthritis with a Syk kinase inhibitor: a twelve-week, randomized, placebo-controlled trial. Arthritis Rheum. 2008;58(11):3309-18. doi:10.1002/art.23992.

48. Weinblatt ME, Schiff MH, Ruderman EM, Bingham CO, 3rd, Li J, Louie J et al. Efficacy and safety of etanercept 50 mg twice a week in patients with rheumatoid arthritis who had a suboptimal response to etanercept 50 mg once a week: results of a multicenter, randomized, double-blind, active drug-controlled study. Arthritis Rheum. 2008;58(7):1921-30. doi:10.1002/art.23493.

49. Westhovens R, Robles M, Ximenes AC, Nayiager S, Wollenhaupt J, Durez P et al. Clinical efficacy and safety of abatacept in methotrexate-naive patients with early rheumatoid arthritis and poor prognostic factors. Ann Rheum Dis. 2009;68(12):1870-7. doi:10.1136/ard.2008.101121.

50. Yazici Y, Curtis JR, Ince A, Baraf H, Malamet RL, Teng LL et al. Efficacy of tocilizumab in patients with moderate to severe active rheumatoid arthritis and a previous inadequate response to disease-modifying antirheumatic drugs: the ROSE study. Ann Rheum Dis. 2012;71(2):198-205. doi:10.1136/ard.2010.148700.

51. Zhang LL, Wei W, Xiao F, Xu JH, Bao CD, Ni LQ et al. A randomized, double-blind, multicenter, controlled clinical trial of chicken type II collagen in patients with rheumatoid arthritis. Arthritis Rheum. 2008;59(7):905-10. doi:10.1002/art.23824.
